# Synthesis, Biological Evaluation and Low-Toxic Formulation Development of Glycosylated Paclitaxel Prodrugs

**DOI:** 10.3390/molecules23123211

**Published:** 2018-12-05

**Authors:** Yukang Mao, Yili Zhang, Zheng Luo, Ruoting Zhan, Hui Xu, Weiwen Chen, Huicai Huang

**Affiliations:** 1 Research Center of Chinese Herbal Resource Science and Engineering, Guangzhou University of Chinese Medicine, Guangzhou 510006, China; mykgzucm@163.com (Y.M.); limeijue520@163.com (Y.Z.); nmrresult@163.com (Z.L.); zhanrt@gzucm.edu.cn (R.Z.); zyfxsherry@gzucm.edu.cn (H.X.); 2 Key Laboratory of Chinese Medicinal Resource from Lingnan (Guangzhou University of Chinese Medicine), Ministry of Education, Guangzhou 510006, China; 3 Joint Laboratory of National Engineering Research Center for the Pharmaceutics of Traditional Chinese Medicines, Guangzhou 510006, China

**Keywords:** paclitaxel, prodrug, solubility, anti-cancer, toxic surfactant

## Abstract

Paclitaxel (PTX) is a famous anti-cancer drug with poor aqueous solubility. In clinical practices, Cremophor EL (polyethoxylated castor oil), a toxic surfactant, is used for dissolution of PTX, which accounts for serious side effects. In the present study, a single glucose-conjugated PTX prodrug (SG-PTX) and a double glucose-conjugated PTX prodrug (DG-PTX) were synthesized with a glycosylated strategy via succinate linkers. Both of the two prodrugs presented significant solubility improvement and drug-like lipophilicities. Compared to DG-PTX, SG-PTX manifested more promising release of the parent drug in serum. A high percentage of PTX released from SG-PTX could be detected after enzymatic hydrolysis of β-glucuronidase. Besides, both of the two prodrugs exhibited effective cytotoxicity against breast cancer cells and ovarian cancer cells, but presented reduced cytotoxicity against normal breast cells. Moreover, SG-PTX manifested impressive solubility in a low toxic formulation (without ethanol) with a different percentage of Cremophor EL. These results indicated that glycosylation is a promising strategy for PTX modification and SG-PTX may be a feasible and potential type of PTX prodrug. In addition, ethanol-free formulation with a low percentage of Cremophor EL might have the potential to develop a safer formulation for further studies of glycosylated PTX prodrugs.

## 1. Introduction

Paclitaxel (PTX) is one of the most famous natural anti-cancer compounds, which is isolated from Taxus brevifolia [1], and effective against various types of cancers [2] including breast cancer, ovarian cancer [3], lung cancer [4], cervical cancer [5] and pancreatic cancer [6]. Although PTX (commercial name is Taxol^®^) can be used for treatment of various cancers, its poor aqueous solubility poses an obstacle in clinical application. A mixture of Cremophor EL (polyethoxylated castor oil) and ethanol (1:1, *v*/*v*) is used as solvent in the commercial PTX injection [7]. Cremophor EL results in hypersensitivity [8],nervous toxicity [9], and inhibition of hepatic elimination [10],and these side effects of Cremophor EL have been shown in dose-dependent manners [11,12]. Thus the product should be diluted by 5 to 20-fold in 5% glucose solution or saline before administration [7]. Hence, to improve aqueous solubility and reduce dosage of Cremophor EL belong to the most important goals for PTX prodrug design.

Hydrophilic modified prodrugs have been synthesized to improve water solubility. An ideal prodrug of PTX should meet two requirements, one is to improve aqueous solubility, the other is to release parent drug to perform its pharmacological effect. Both small molecular and macromolecular modifications have been reported for PTX prodrugs design. On the one hand, PTX prodrugs modified with small hydrophilic molecules such as morpholino [13] and folic acid [14] have been reported, these prodrugs present better aqueous solubility and potential anti-cancer effects. On the other hand, PTX prodrugs with macromolecule modification have been proposed. In addition to aqueous solubility improvement, some prodrugs manifest special effects. For example, polyethylene glycol (PEG)-PTX prodrug presents lower toxicity and higher safety [15], polystyrene-co-maleic acid (PSMAC)-PTX prodrug manifests pH sensitivity [16], prodrug consisting of PTX and cell penetrating peptide tLyP-1 is endowed with a potential tumor targeting effect [17].

Glycosylation is one of the most feasible strategies of prodrug design. Glucose is a non-toxic and highly hydrophilic molecule, thus conjugating the parent drug with a glucose moiety can improve aqueous solubility without additional toxic effect. After being modified with glucose analogs, PTX prodrugs manifest promising cytotoxicity against cancer cells, and they can be transported into cells on the membrane surface through Glucose transporters (GLUTs) [18], suggesting that glycosylated PTX prodrugs may be absorbed by cancer cells via the transporting mechanism of glucose.

Linkers should be used for linking the parent drug and the glycosylated structure. The anti-cancer effect is affected by the type of linkers; ester bonds and ether bonds are the most commonly used linkers in PTX prodrugs design. Esterification is a common approach for changing solubility and lipophilicity of a compound with hydroxyl groups. It has been reported that PTX prodrugs with linker as ester bonds present better cytotoxicity and lower IC_50_ (50% inhibitory concentration) against different types of cancer cells than that of PTX glycosyl prodrugs with linker as ether bonds, indicating that glycosylated PTX prodrug with an ester bond present better in vitro anti-cancer effect [18]. What is more, β-glucuronidase, a lysosomal glycosidase has been used as a cancer marker, catalyzing the degradation of glycosaminoglycans which contain glucuronic acid [19,20]. Therefore, we hypothesize that the glycosylated prodrugs can take advantage of β-glucuronidase activity and release parent drug into the blood.

**Figure 1:**
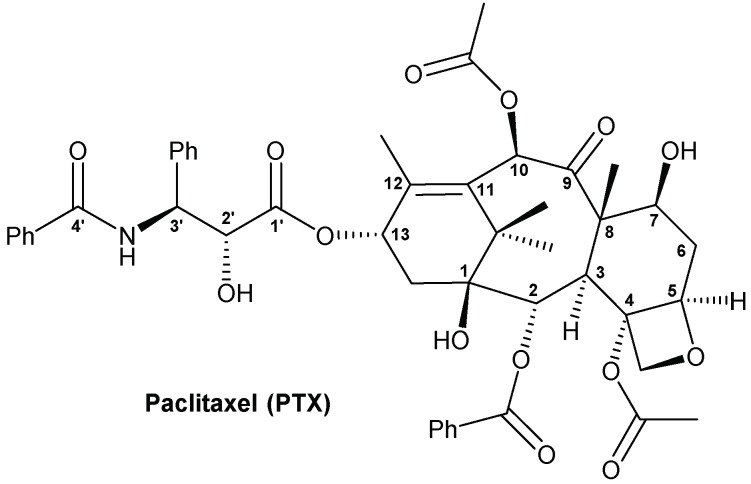
Chemical structure of paclitaxel.

The chemical structure of PTX is shown in Figure 1. Common-used positions for modification of PTX are 2′-OH at the side chain and 7-OH at the four-ring structure. Previous studies have presented prodrugs with glycosylation at the 7-OH position of PTX [21,22]. However, prodrugs with glucose conjugates at the 2′-OH position and double glycosylation (at both 2′-and 7-OH) lack published articles. Moreover, no suitable formulation of glycosylated PTX prodrug has been reported yet. Thus, the aim of this study was to design and synthesize two glycosylated PTX prodrugs, and evaluate their in vitro anti-cancer effects, aqueous solubilities, drug release, enzymatic hydrolysis, and formulation development. Both of the two prodrugs linked with glucose via a succinate linker at the 1-OH of glucose, but one prodrug is modified at only the 2′-OH of PTX (single glucose-conjugated Paclitaxel, SG-PTX), the other prodrug is modified at both the 2′-OH and 7-OH positions of PTX (double glucose-conjugated Paclitaxel, DG-PTX).

## 2. Results

### 2.1. Synthesis of SG-PTX and DG-PTX

**Scheme 1:**
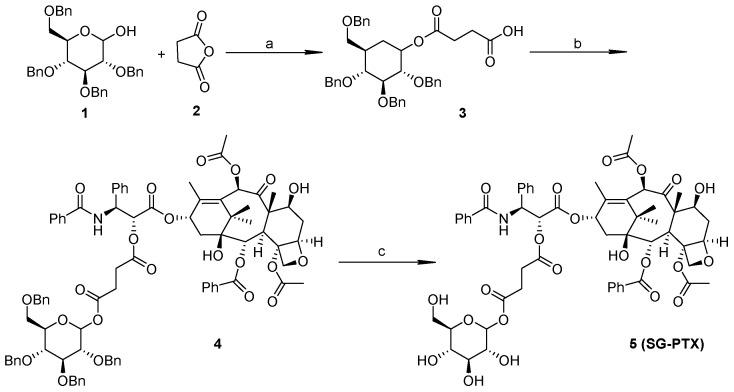
Synthesis of single glucose-conjugated paclitaxel (SG-PTX). Reagents and conditions: (a)DMAP, DCM, 16 h, 99%; (b) PTX, DMAP, DCC, THF, 24 h, 57%; (c) H_2_, Pd/C, MeOH, AcOH, 24 h, 75%. “DMAP” stands for 4-dimethylaminopyridine, “DCM” for dichloromethane, “PTX” for paclitaxel, “DCC” for dicyclohexylcarbodiimide and “THF” for tetrahydrofuran.

**Scheme 2:**
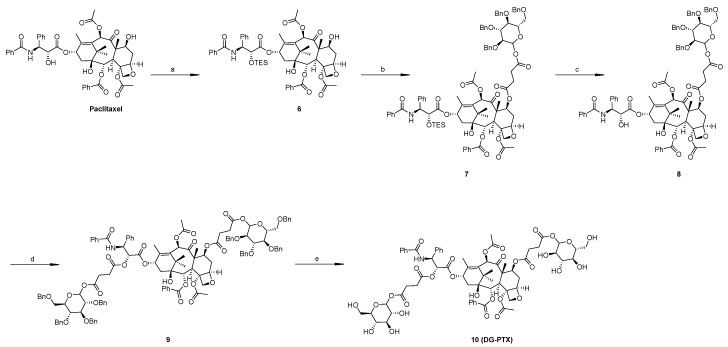
Synthesis of double glucose-conjugated paclitaxel (DG-PTX). Reagents and conditions: (a) TESCl, imidazole, 4 h, 66%; (b) compound **3**, DMAP, DCC, THF, 24 h, 53%; (c) TBAF, THF, 6 h, 66%; (d) compound **3**, DMAP, DCC, THF, 24 h, 55%; (e) H_2_, Pd/C, AcOH, 24 h, 85%. “TESCl” stands for chlorotriethyl silane and “TBAF” for tetrabutylammonium fluoride.

Synthesize routes of SG-PTX and DG-PTX are shown in Scheme 1 and Scheme 2, respectively. Both of the two prodrugs were characterized with ^1^H-NMR, ^13^C-NMR, and MS.

Compound **1** is vital for the synthesis of SG-PTX and DG-PTX. Except for the 1-OH position, all other hydroxyl groups of glucose are protected by benzyl groups, so this position provides a certain reactive position for succinate anhydride. When the oxygen heterocyclic ring of succinate anhydride is opened, a carboxyl group is exposed for esterification (compound **3**).

To synthesize SG-PTX, compound **3** was used for modification of PTX, esterification was conducted between the carboxyl group and 2′-OH of the PTX, then hydrogenation was used to deprotect the benzyl groups of glucose.

Synthesis of DG-PTX is more complicated because 2′-OH at the C-13 side chain is more active than 7-OH at the four-ring structure. Previous studies demonstrated that before modification at 7-OH, protection with the triethylsilane (TES) group at 2′-OH is necessary [23,24], so esterification at 7-OH was conducted after protection at 2′-OH. After that, the TES group was deprotected by tetrabutylammonium fluoride (TBAF) and 2′-OH was modified with compound **3** as SG-PTX. Lastly, hydrogenation was used to deprotect benzyl group.

### 2.2. Solubility Assays of SG-PTX and DG-PTX

**Figure 2:**
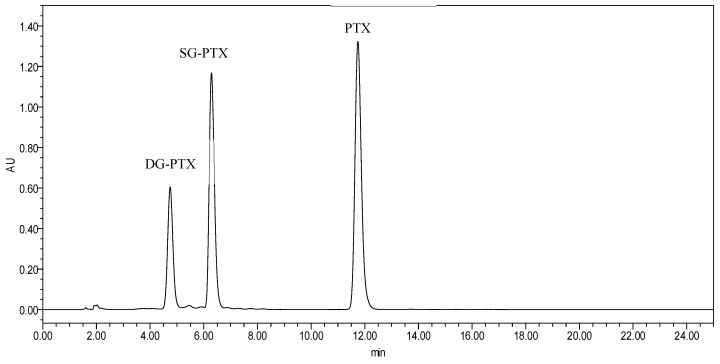
HPLC chromatography of PTX, SG-PTX, and DG-PTX.

HPLC chromatography of PTX and PTX prodrugs is shown in Figure 2.

The retention behaviors of PTX and PTX prodrugs reveal different polarities of these compounds. In reverse-phase HPLC determination, compounds with larger polarity manifest shorter retention time. Approximately, the retention time was 11.9 min for PTX, 6.3 min for SG-PTX, and 4.9 min for DG-PTX, indicating that the chemical structure of the substituent moiety plays an important role in the polarity of PTX prodrugs. SG-PTX exhibited to be more polar than PTX because of the polar glucose moiety. Similarly, DG-PTX exhibited the largest polarity among the compounds as a result of its double substituent glycosylated moiety.

Solubility of PTX and PTX prodrugs is shown in Table 1, linearity calibration was conducted before HPLC determination (Figures S1 and S2).

Concentrations of PTX in the three medias were lower than the LOD (Limit of Detection) of the HPLC method, but previous study pointed out that the aqueous solubility of PTX is 0.4 μg/mL [25]. As is shown in Table 1, the aqueous solubility of PTX increased from 0.4 μg/mL to 17.89 μg/mL with glucose conjugation, which was 49.8 times that of the parent drug. The aqueous solubility of DG-PTX climbed to 38.97 μg/mL, which was 97.4 times of PTX. Both SG-PTX and DG-PTX are more soluble than the parent drug in these medias, suggesting that solubility of PTX could be improved significantly with glycosylation strategy. The solubility increased with the number of glycosylated moieties and DG-PTX was the most hydrophilic, which is attributed to the hydrophilicity of glucose. Coincidently, DG-PTX is the most soluble among the three compounds in these injective medias. Moreover, there is no significant difference between the three medias.

The strongly hydrophobic character of PTX accounts for the very poor aqueous solubility and concentrations of PTX in the different mediums were lower than the LOD. The significant solubility improvement indicated that the aqueous solubility of the PTX prodrugs improves with the number of glycosylated moieties. In the medicinal chemical area, a compound with solubility lower than 10 μg/mL is considered to be of low solubility, 10–100 μg/mL as moderately soluble and a compound with solubility higher than 100 μg/mL is considered to be highly soluble [26]. The solubility improvement indicated that the glycosylation strategy can boost paclitaxel from poorly soluble to moderately soluble, which enhances the potential for further development of PTX prodrugs.

### 2.3. Determination of Partition Coefficient (Log P) of SG-PTX and DG-PTX

Determination of log P is another way of evaluation of lipophilicity of the prodrugs. Determination result of log P of PTX prodrugs is shown in Table 2.

Lipophilicity affects the physicochemical and physiological properties of a compound such as the aqueous solubility and permeability through biological membranes. It is described in most cases as the partition between the hydrophilic phase and the hydrophobic phase [28]. Log P is in the optimum range for polar and lipid bilayer permeability (0–3), compound with a higher log P (>3) is defined to be non-polar and has poor aqueous solubility [29]. Compared to high log P of PTX, both the prodrugs possessed more suitable drug-like properties as consequences of lipophilicity, indicating that glycosylation turns PTX into a more hydrophilic form.

### 2.4. Parent Drug Release Assays of SG-PTX and DG-PTX

**Figure 3:**
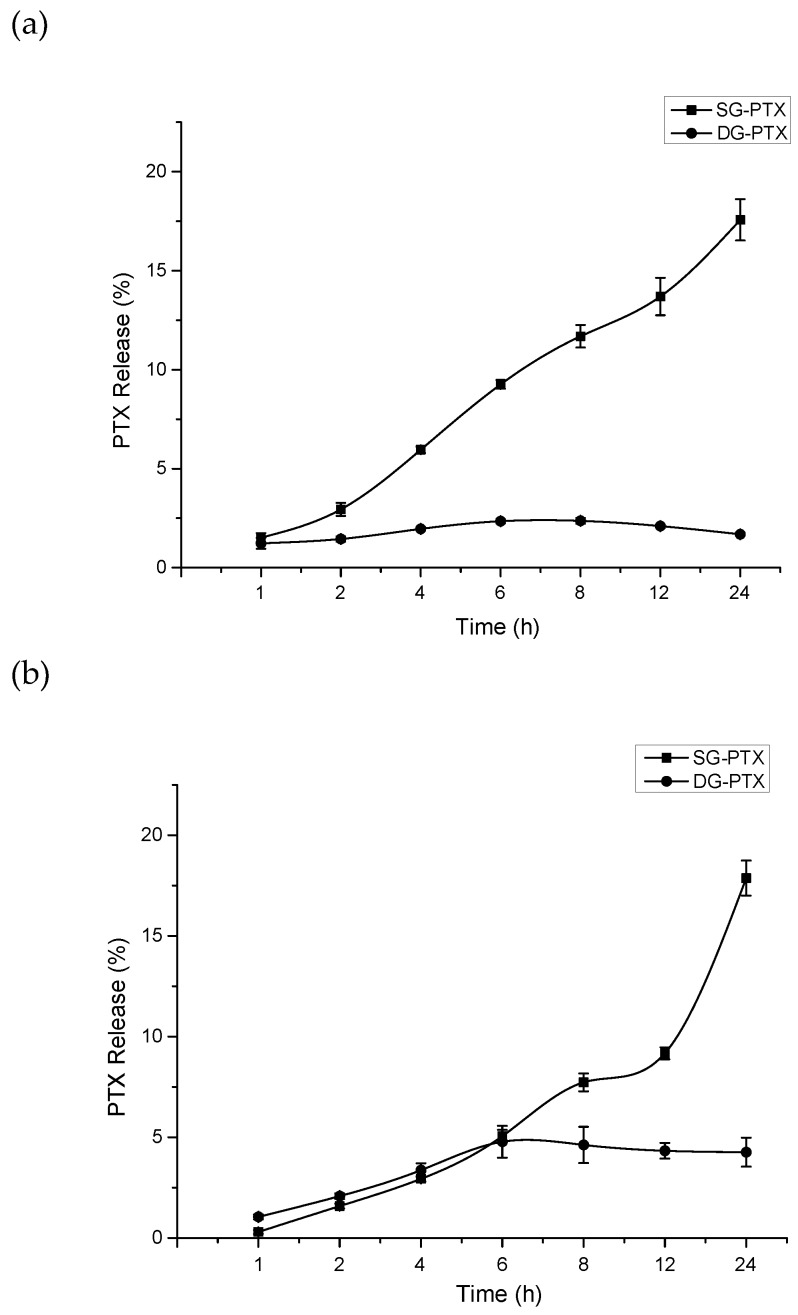
Free PTX released from the prodrugs in serum. (**a**) PTX released from SG-PTX and DG-PTX in FBS, (**b**) PTX released from SG-PTX and DG-PTX in RS. All values are expressed as mean ± SD (n = 5). “FBS” stands for fetal bovine serum and “RS” for rabbit serum.

Parent drug release is an important factor for prodrug design, thus we studied and compared the amount of PTX release of the two prodrugs in fetal bovine serum (FBS) and rabbit serum (RS) at different times, the results are shown in Figure 3.

The drug release pattern manifested obvious differences between the two prodrugs. In FBS, with time, the percentage of PTX released from SG-PTX increased and climbed to 17.57% after 24 h incubation. On the contrary, DG-PTX showed only 1.69% of parent drug released after 24 h incubation. PTX released from DG-PTX showed no relationship with time passing. In addition, although the percentages of PTX release of the two prodrugs were similar before 6 h in RS, PTX released from SG-PTX increased and climbed to 17.87% after 24 h incubation in RS while DG-PTX released only 4.26% of the parent drug after 24 h incubation. The result indicates that free PTX could be released from glycosylated prodrugs, and the succinate linker could be cleaved in serum. Moreover, SG-PTX manifested better release of PTX and its ester bond at 2′-OH could be easily cleaved. However, DG-PTX showed poor release of PTX, this phenomenon may be due to the abundance of ester bonds of DG-PTX and its steric hindrance of 7-OH; the ester bond at 2′-OH could be cleaved but 7-OH remained stable in FBS and RS, which accounts for the low concentration of free PTX.

Besides, the mechanism of the prodrugs breaks the ester bonds and releases parent drugs to present cytotoxic effects. Therefore, the better in vitro anti-cancer effect of SG-PTX may be a consequence of its promising release of free PTX.

PTX could be released from SG-PTX in both FBS and RS and the content of PTX improved with time ongoing. This result indicates that the succinate linker on 2′-OH of SG-PTX can be cleaved in blood of different species and PTX can be released to present its pharmacological effect. The mechanism of PTX release may be the esterase effect induced by glucuronidase in mammal serum, and the ester bonds are broken by glucuronidase.

### 2.5. Enzymatic Hydrolysis of SG-PTX and DG-PTX

**Figure 4:**
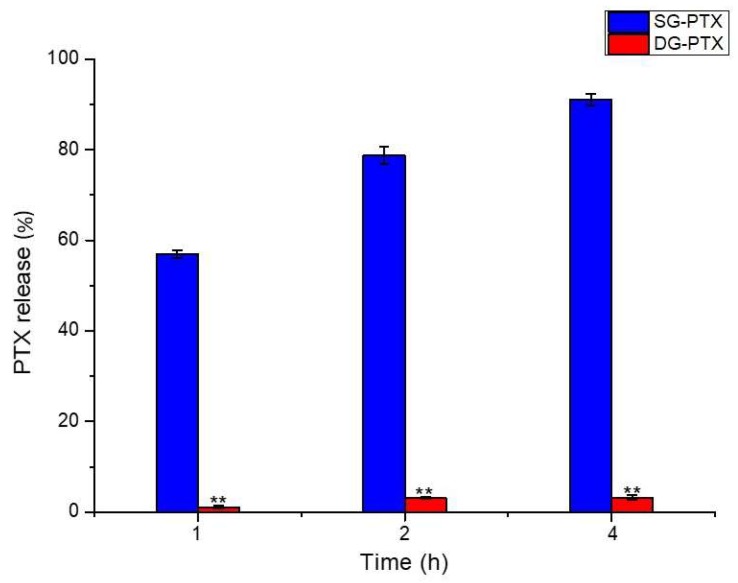
Enzymatic hydrolysis of SG-PTX and DG-PTX. All values are expressed as mean ± SD (n = 5). Compared to SG-PTX group, ** *p* < 0.01.

Previous study suggested that glycan-based PTX prodrugs with succinic acidic linkers could be cleaved by β-glucuronidase and PTX could be released [18]. To further clarify the mechanism of PTX released from the prodrugs, in vitro enzymatic hydrolysis of PTX prodrugs was evaluated in solution contained β-glucuronidase and the result is shown in Figure 4.

Hydrolysis behavior in solution of β-glucuronidase presented a significant difference between the two prodrugs. The percentage of PTX release from SG-PTX increased dramatically to 56.95% in 1 h and climbed to 91.01% after 4 h incubation. Nevertheless, compared to SG-PTX, very little PTX was released from DG-PTX, only 1.09% of parent drug was released in 1 h and 3.15% after 4 h incubation. According to the hydrolysis result, it can be inferred that the ester bond at 2′-OH is able to be cleaved by β-glucuronidase and free PTX can be released from SG-PTX. The hydrolytic product of DG-PTX may be a PTX derivative with a glucose moiety at 7-OH, via a succinic ester linker. Low percentage of PTX released from DG-PTX may be a consequence of the steric hindrance of the ester bond at 7-OH. Previous study demonstrated that the ester bond at 7-OH causes steric hindering, is less reactive, and unable to interact with esterase [30,31], which leads to difficulty for the ester bond cleaving at 7-OH and results in poor quantity of free PTX.

### 2.6. In Vitro Anti-Cancer Effect Evaluations of SG-PTX and DG-PTX

**Figure 5:**
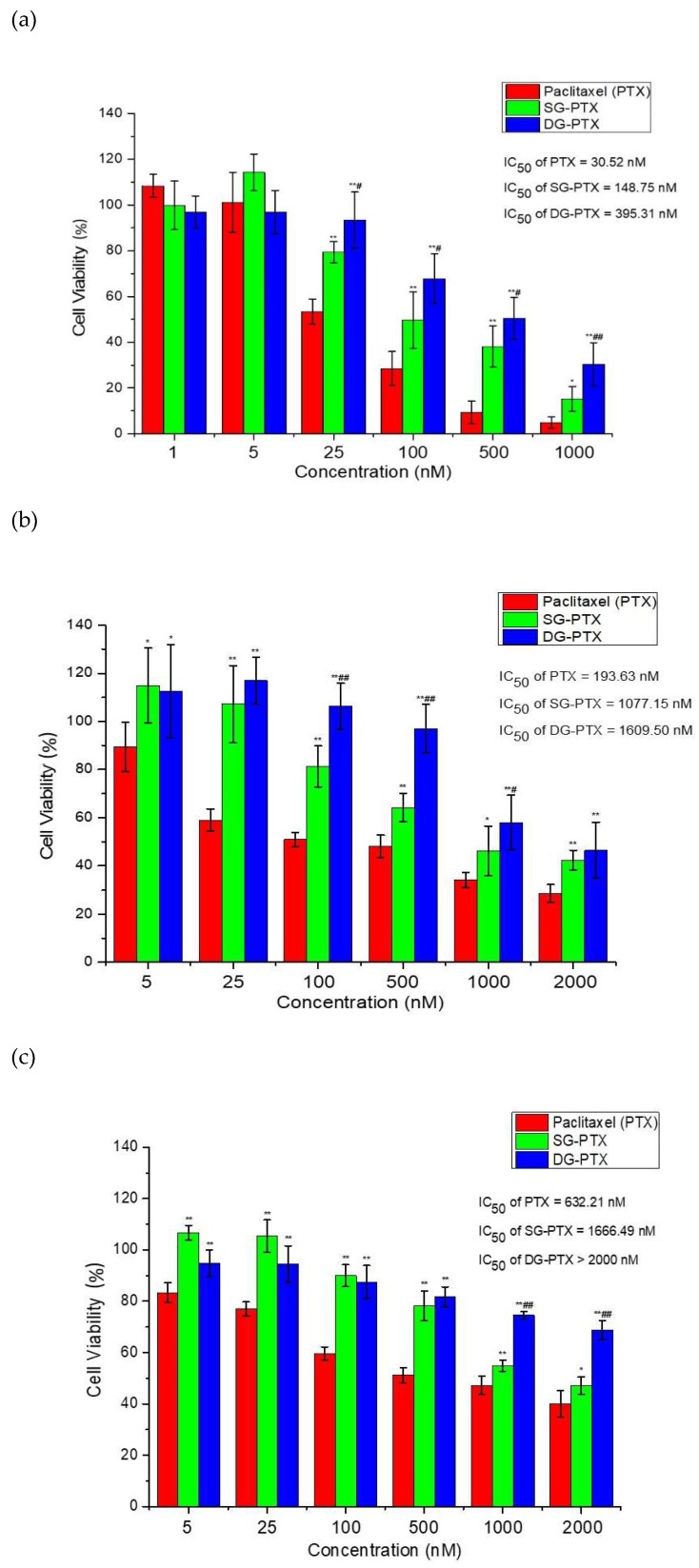
Cytotoxicity of PTX and PTX prodrugs against cancer cells and normal cells. (**a**) breast cancer cells MDA-MB-231, (**b**) ovarian cancer cells A2780, (**c**) normal breast cells Hs578Bst. All values are expressed as mean ± SD (n = 6). Compared to PTX group, * *p* < 0.05, ** *p* < 0.01; compared to SG-PTX group, ^#^ *p* < 0.05 ^##^ *p* < 0.01.

In vitro anti-cancer effects against cancer cells and safety to normal cells of the prodrugs were evaluated by cytotoxicity. Cytotoxicity against breast cancer cells MDA-MB-231, ovarian cancer cells A2780, and normal breast cells Hs578Bst were tested via MTT assays, the cells were treated with PTX, SG-PTX, and DG-PTX and incubated for 72 h, then cell viability was measured with OD detection, the results are shown in Figure 5.

Viability of cancer cells treated with PTX and PTX prodrugs exhibited dose-effect dependent relationships, cytotoxicity increased with the concentration improvement of PTX and PTX prodrugs. Cell viability decreased when the concentration of PTX and PTX prodrugs was higher than 5 nM, suggesting cell growth inhibition increased with drug concentration improvement. IC_50_ values for PTX and PTX prodrugs showed 30.52 nM for PTX, 148.75 nM for SG-PTX, and 395.31 nM for DG-PTX against MDA-MB-231 cells. Moreover, IC_50_ values were 193.63 nM for PTX, 1077.15 nM for SG-PTX, and 1609.50 nM for DG-PTX against A2780 cells. Although the cytotoxicity of SG-PTX and DG-PTX was lower than the parent drug, they still presented promising anti-cancer effects in vitro. Previous studies have pointed out that it is acceptable to have higher IC_50_ for prodrugs than the free parent drug [32,33,34] because the free form of the parent drug must be released from the prodrug to exert a pharmacological effect.

Besides, IC_50_ values were 632.21 nM for PTX and 1666.49 nM for SG-PTX. The IC_50_ value was higher than 2000 nM for DG-PTX against Hs578Bst.The low toxicity of the prodrugs in normal breast cells indicated that the glucose-ester conjugations could reduce the toxicity of PTX and improve the safety of PTX prodrugs used for potential clinical practice.

**Figure 6:**
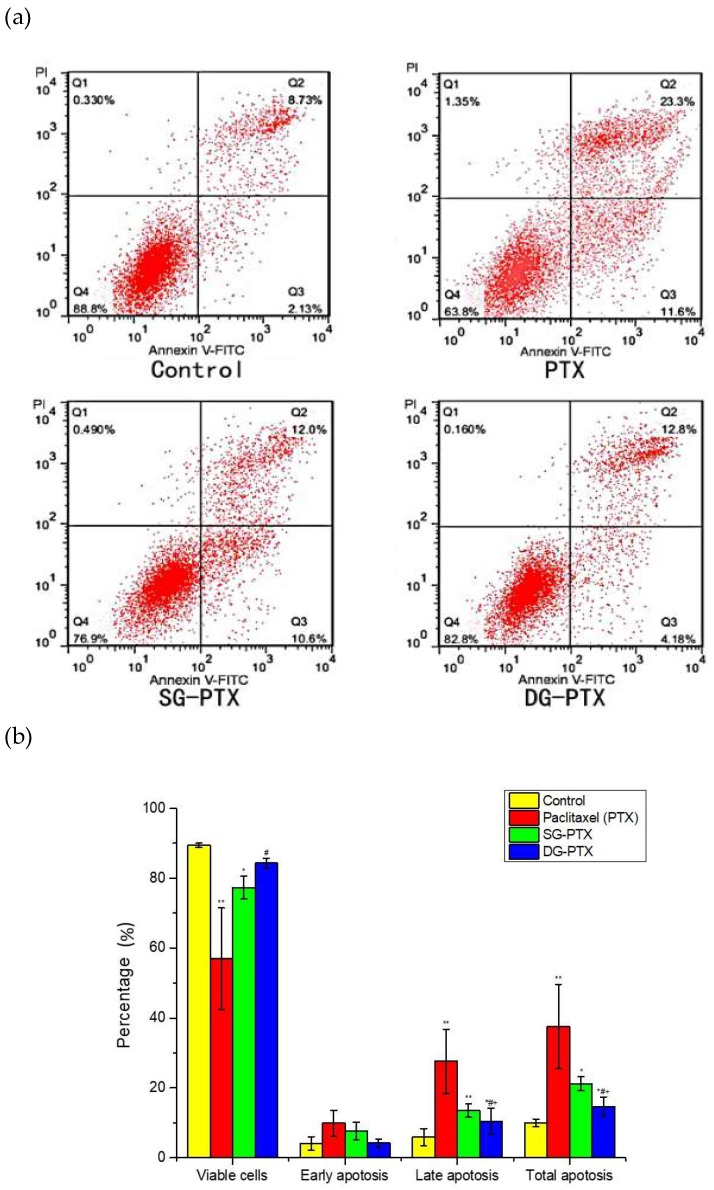
Cell apoptosis inducing effect of PTX and PTX prodrugs on MDA-MB-231 cells. (**a**) Apoptosis of MDA-MB-231, (**b**) Quantification of apoptosis data. All values are expressed as mean ± SD (n = 3). Compared to control group, * *p* <0.05, ** *p* < 0.01; compared to PTX group, ^#^ *p* < 0.05; compared to SG-PTX group, ^+^ *p* < 0.05.

Cell apoptosis was tested by an Annexin V-FITC/PI staining assay and analyzed by flow cytometry, the result is illustrated in Figure 6. According to Figure 6a, the four regions represent different cell stages. Q1 represents necrotic cells (Annexin V+/PI–), Q2 and Q3 represent late apoptotic cells (Annexin V+/PI+) and early apoptotic cells (Annexin V+/PI–), and Q4 represents normal cells (Annexin V–/PI–). From Figure 6b, it can be seen that total percentage of apoptotic cells (percentage of total apoptosis = late apoptosis + early apoptosis) sharply increased to 37.5% and 21.23% after treatment with PTX and SG-PTX, indicating that PTX and SG-PTX manifested a significant apoptosis inducing effect against MDA-MB-231 cells. In addition, the percentage of apoptotic cells induced by DG-PTX was 14.6%. In the control group, 89.4% of the cells were viable. The apoptosis results reveal that both SG-PTX and DG-PTX induce apoptosis in breast cancer cells while the apoptosis inducing effect of SG-PTX is stronger than DG-PTX.

In short, SG-PTX was selected for formulation development based on the results of cytotoxicity, apoptosis inducing effect, drug release, and enzymatic hydrolysis.

### 2.7. Solubility of SG-PTX at Different pH Conditions

**Figure 7:**
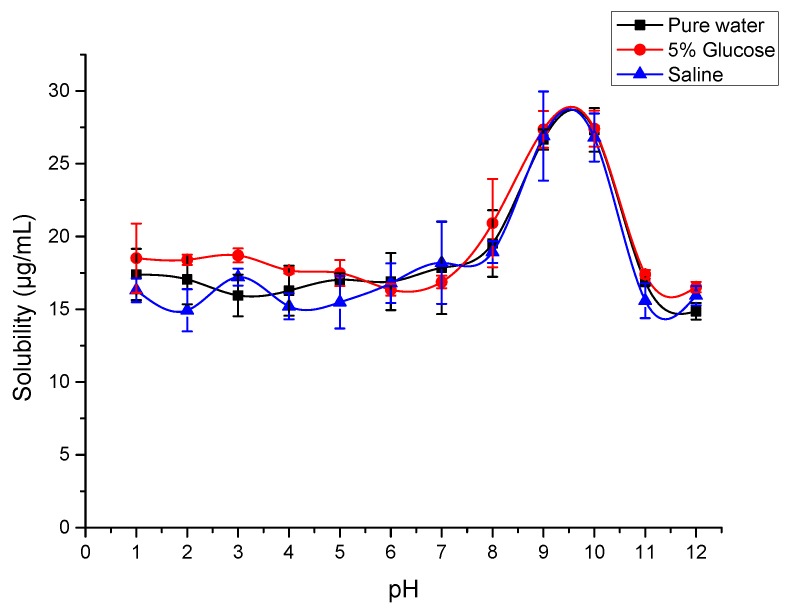
Solubility of SG-PTX at different pH conditions in three common media. All values are expressed as mean ± SD (n = 4).

Different body fluid maintains different pH conditions in the human body [26], the solubility of SG-PTX at different pH conditions was tested and the result is shown in Figure 7.

The result suggests that the solubility of SG-PTX can be affected by pH levels. In water and two common media used for PTX injection in clinical practices, the solubility of SG-PTX increased when the pH was higher than 8, and climbed to about 27 μg/mL at pH 9 and pH 10, then decreased when the pH was higher than 10, implying that the solubility of SG-PTX is affected by pH change. What is more, the solubilizing behaviors of SG-PTX in the three common media were similar. Structural features of SG-PTX have little influence on the solubility of the compound at different pH conditions. The absence of an ionizable functional group in SG-PTX causes its solubility to be similar at different pHs in the respective media. SG-PTX does not contain an ionizable functional group that allows pH alternation and salt formation to improve its solubility. Moreover, SG-PTX showed no significant change at the pH level of 1 to 8, indicating that the solubility of SG-PTX can remain stable at pH conditions of the human body, even at the pH of cancer cells [35]. The changeless solubilizing behavior implied that SG-PTX may not be ionized and may maintain its free form in the human body, which is consistent with PTX.

### 2.8. Formulation Development of SG-PTX

Solubility of SG-PTX in various formulations was tested, SG-PTX was added into formulations with different proportion (*v*/*v*) of water and surfactants. These solubility tests were carried out according to the same protocol as aqueous solubility assays.

**Figure 8:**
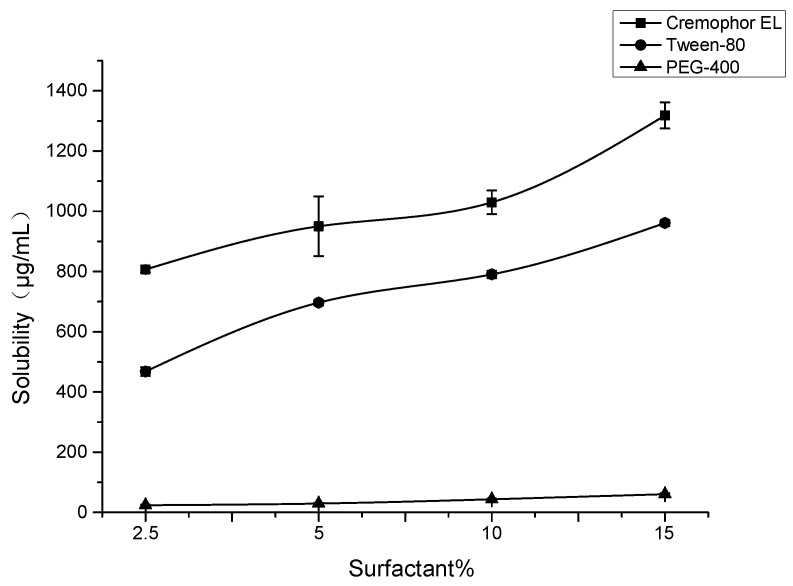
Solubility of SG-PTX in various formulations with different proportion of surfactants (Cremophor EL, Tween-80, and PEG-400). All values are expressed as mean ± SD (n = 4).

Different surfactants containing various proportion of water and surfactants were evaluated to screen the best surfactant for improvement of SG-PTX solubility. As is shown in Figure 8, both Cremophor EL and Tween-80 could improve the solubility of SG-PTX and the solubility improvement presented dose-effect dependence. When the percentage of surfactant was 2.5%, the solubility of SG-PTX was 806.67 μg/mL in Cremophor EL and 467.70 μg/mL in Tween-80. When the percentage of surfactant was 15%, the solubility of SG-PTX climbed to 1318.14 μg/mL in Cremophor EL and 961.08 μg/mL in Tween-80. However, when PEG-400 was used, the solubility of SG-PTX was the lowest and presented poor solubility improvement.

Among the three surfactants, Cremophor EL and Tween-80 were able to significantly increase the solubility of SG-PTX while PEG-400 presented poor solubility improvement of SG-PTX. To compare with Tween-80, Cremophor EL manifests a better solubility increase effect in the same composition of formulation. The promising solubility improvement of Cremophor EL is due to its emulsifying function, the hydrophobic drugs are wrapped in a microemulsion, but the hydrophilic groups are lined on the surface of the microemulsion, which causes solubility improvement. In clinical practice, when Cremophor EL was 2.5% in the Taxol^®^ injection, the concentration was 300 μg/mL [36], which is the least dosage for the paclitaxel injection. Interestingly, the solubility of SG-PTX was 806.67 μg/mL in the same proportion of Cremophor EL, suggesting that the dosage of Cremophor EL could be reduced to achieve the same solubility. Moreover, all formulations contain no ethanol, indicating that SG-PTX can achieve a promising solubility in ethanol-free formulations, which is safer than the commercial solvent in clinical application.

## 3. Discussion

The development of prodrugs follows similar research and regulatory paths to a typical small-molecule new chemical entity. A potential prodrug requires activation to release its parent drug. As described previously, glycosylated PTX prodrugs (via an ether bond as linker) have been proposed, which enhance aqueous solubility and maintain competitive cytotoxicity [25]. Results from the previous study suggest that glycosylation is a promising strategy for PTX prodrug design.

The present study synthesized two PTX prodrugs with glycosylated strategy, both of the two prodrugs improved aqueous solubility significantly, and presented more hydrophilic properties such as lower log P, indicating that the prodrugs possess better drug-like lipophilicity in comparison to PTX.

Parent drug release plays a critical role in prodrug design. However, the determination of free PTX released from glycosylated prodrug lacks published studies. As is illustrated in the present study, the free form of PTX can be released from the prodrugs in both FBS and RS. The percentage of free PTX released from SG-PTX is much higher than DG-PTX, indicating that SG-PTX can release PTX in serum.

β-glucuronidase degrades glycosaminoglycans which contain glucuronic acid and it is overexpressed in a variety of cancers such as neoplasm of breast and larynx [37], liver tumor [20], and colon cancer [38], so it is used as a biomarker of cancer [19]. Furthermore, β-glucuronidase presents a prodrug-releasing function [39,40] and it activates low-toxic prodrugs to become highly cytotoxic agents specifically at the tumor site [41]. In the present study, hydrolysis of SG-PTX could be facilitated by β-glucuronidase, more than 90% of the parent drug was released by the action of β-glucuronidase within 4 h. On the contrary, only approximately 3% of PTX was released from DG-PTX after incubation with β-glucuronidase. This result indicated that the ester bond of SG-PTX is labile and is prone to enzymatic hydrolysis. It can be inferred that ester bonds at 2′-OH may be easily cleaved by enzymatic hydrolysis so that PTX can be readily released from SG-PTX. However, the ester bond at 7-OH may remain stable after incubation with β-glucuronidase, which might be the result of steric hindrance and low reactive activity of 7-OH. β-glucuronidase is abundant in the human gastrointestinal tract [42] and the enzyme can be identified from the human gut bacterium *Bacteroides uniformis* [43]. Except for glucuronic-acidic compounds, β-glucuronidase is capable of hydrolyzing a series of sugar-acid containing substrates such as derivatives of galacturonic acid, iduronic acid, and mannuronic acid [43]. Besides, low aqueous solubility is one of the most important obstacles of oral administrations of PTX [44]. Unfortunately, usage of Cremophor EL decreases the absorption of PTX from the gut [45], which forms a contradiction between aqueous solubility and absorption. In the present study, SG-PTX could be hydrolyzed by β-glucuronidase and release PTX, which also exhibited higher aqueous solubility and reduction of Cremophor EL dosage. SG-PTX may release PTX in the human gut because of the abundance of β-glucuronidase. Moreover, the solubility improvement and dosage reduction of Cremophor EL may boost absorption of the parent drug in the gut. Therefore, glycosylated prodrug may be a potential form for oral administration of PTX.

The prodrugs also manifested effective cytotoxicity and apoptosis inducing effect against breast cancer cells, cytotoxicity of the prodrugs was maintained at nM level. On the other hand, the prodrugs manifested significant apoptosis inducing effects. In short, SG-PTX presented a stronger anti-cancer effect than DG-PTX. The differences in cytotoxicity and apoptosis inducing effect may be consequences of difference of lipophilicity, chemical structure, and parent drug release. PTX has to cross the biological membrane through passive transport because PTX has no specific receptor or carrier on the cell membrane. Thus, lipophilicity of PTX helps the drug to pass through the cell membrane and present an anti-cancer effect. Although the in vitro anti-cancer effects of the prodrugs are not as strong as PTX, the cytotoxicity and apoptosis inducing effect of the prodrugs are still competitive. The pharmacological differences are acceptable in view of the merits of the prodrugs, such as aqueous solubility improvement and dosage reduction of toxic surfactants.

Briefly, SG-PTX was found to be suitable for PTX prodrug design. It has been reported that the ester prodrug of a compound can be an effective way to optimize the drug delivery property and convert the prodrug to its parent form [46,47]. From the results of the present study, it can be supposed that esterification is a viable strategy for PTX prodrug design, but the reactive position chosen for esterification needs to be considered.

Prodrug strategy can be used as an alternative form to eliminate the toxic surfactant-based vehicle [48]. In the present study, ethanol-free formulations were developed. A variety of commercial surfactants were used to screen a suitable formulation for SG-PTX and high solubility of SG-PTX was observed in the formulation with a low proportion of Cremophor EL. Based on the dose-dependent toxicity of Cremophor EL, reducing the proportion of Cremophor EL in the formulation can alleviate side effects. The high solubility of SG-PTX in the lower toxic formulation implied that SG-PTX may be a safer alternative to PTX.

Result of the present study indicated that glycosylation strategy is viable for PTX prodrug design and SG-PTX is a promising type of PTX prodrug. Based on these data, further in vivo pharmacological evaluation should be carried out to better confirm the anti-cancer activity of SG-PTX, with the aim of discovering the PTX prodrug that may be advanced to the clinical stages of development.

## 4. Materials and Methods

### 4.1. General

Paclitaxel, succinate anhydride, 2,3,4,6-Tetra-*O*-benzyl-d-glucose, succinic anhydride, 4-dimethylaminopyridine (DMAP), dicyclohexylcarbodiimide (DCC), palladium on 10% activated carbon (10% Pd/C), chlorotriethylsilane (TESCl), tetrabutylammonium fluoride (TBAF), dichloromethane (DCM), tetrahydrofuran (THF), methanol (MeOH), acetic acid (AcOH), n-hexane (Hex), and ethyl acetate (EA) were purchased from Energy Chemicals (Shanghai, China). Cremophor EL, Tween-80 and PEG-400 were purchased from Macklin (Shanghai, China).

3-(4,5-dimethyl-2-thiazolyl)-2,5-diphenyl-2-*H*-tetrazolium romide (MTT), dimethyl sulfoxide (DMSO), and β-glucuronidase were purchased from Sigma Aldrich (St. Louis, MO, USA). Leibovitz’s medium (L-15 medium), fetal bovine serum (FBS), and Hank’s balanced salt solution (HBSS) were purchased from Gibco (New York, NY, USA), rabbit serum (RS) was purchased from BestBio (Shanghai, China).

NMR spectra were recorded on a Bruker AVANCE III HD 400MHz Digital NMR Spectrometer (Karlsruhe, Germany). ESI mass spectra were recorded on a Thermo Scientific TSQ Quantum^TM^ Access MAX Mass Spectrometer (Waltham, MA, USA). Optical Density (OD) of MTT assay was measured with a Bio-rad iMark 96-well plate reader (Hercules, CA, USA).

### 4.2. Synthesis of SG-PTX

Synthesis of succinyl benzyl glucose (**3**): the mixture of 2,3,4,6-Tetra-*O*-benzyl-d-glucose (1.62 g, 3 mmol), succinic anhydride (900.18 mg, 9 mmol), and DMAP (366.5 mg, 3 mmol) in DCM (8 mL) were stirred at r.t. for 16 h, then extracted with 0.1 M HCl, dried over MgSO_4_ and concentrated. The residue was purified by silica gel chromatography (Hex:EA = 4:1), and the solvent was removed under pressure to produce a colorless oil **3** (1.99 g, yield 100%). ^1^H-NMR indicated that chemical shift of compound **3** was consistent with reference [18].

Synthesis of C2′-benzyl glycosylated paclitaxel (**4**): the mixture of paclitaxel (1.11 g, 1.3 mmol), DMAP (79.4 mg, 0.65 mmol) and DCC (536.5 mg, 2.6 mmol) in THF (4 mL) were added to a solution of compound **3** (1.25 g, 1.95 mmol) in THF (4 mL). After stirring at r.t. at N_2_ atmosphere for 24 h, and concentrating under pressure, the residue was purified by silica gel chromatography (Hex:EA = 2:1), and the solvent was removed under pressure to produce a white solid **4** (1.09 g, yield 57%). ^1^H-NMR (400 MHz, Chloroform-d) δ 8.15 (d, *J =* 7.7 Hz, 2H), 7.76 (d, *J =* 7.5 Hz, 2H), 7.64–7.21 (m, 30H), 7.24–6.92 (m, 5H), 6.37–6.15 (m, 3H), 5.95 (ddd, *J =* 22.1, 9.1, 3.3 Hz, 1H), 5.69 (d, *J =* 7.1 Hz, 1H), 5.50 (dd, *J =* 10.4, 3.2 Hz,1H), 4.99–4.06 (m, 13H), 3.93–3.47 (m, 7H), 2.73 (qd, *J =* 18.4, 16.6, 8.2 Hz, 4H), 2.61–2.49 (m, 2H), 2.48–2.32 (m, 4H), 2.21 (s, 4H), 2.04 (s, 1H), 1.98–1.84 (m, 6H), 1.78 (s, 4H), 1.68 (s, 4H), 1.30–1.04 (m, 9H).

Synthesis of C2′-single glycosylated paclitaxel (**5**, SG-PTX): compound **4** (1.09 g, 0.74 mmol) was dissolved in a mixture of MeOH (2 mL) and AcOH (4 mL), then Pd/C (472.5 mg, 4.44 mmol) was added. The reaction was stirred at r.t. in a H_2_ atmosphere for 24 h. After filtering and concentration, the residue was purified by silica gel chromatography (EA:MeOH = 10:1), and the solvent was removed under pressure to produce a white solid **5** (618.2 mg, yield 75%). ^1^H-NMR (400 MHz, DMSO-*d*_6_) δ 9.23 (d, *J =* 8.7 Hz, 1H), 8.02–7.97 (m, 2H), 7.89–7.83 (m, 2H), 7.74 (dd, *J =* 8.5, 6.1 Hz, 1H), 7.66 (t, *J =* 7.5 Hz, 2H), 7.59–7.43 (m, 8H), 7.21 (p, *J =* 4.4 Hz, 1H), 6.31 (s, 1H), 5.95 (d, *J =* 3.5 Hz, 1H), 5.85 (q, *J =* 7.9, 7.1 Hz, 1H), 5.58 (td, *J =* 8.6, 5.6 Hz, 1H), 5.46–5.33 (m, 2H), 5.11 (t, *J =* 5.6 Hz, 1H), 5.04 (dd, *J =* 10.2, 5.3 Hz, 1H), 4.94 (dd, *J =* 17.3, 6.2 Hz, 3H), 4.65 (d, *J =* 3.2 Hz, 1H), 4.49 (t, *J =* 5.8 Hz, 1H), 4.17 *J* = 4.08 (m, 1H), 4.07–3.97 (m, 4H), 3.64–3.54 (m, 2H), 3.48 (ddd, *J =* 18.3, 10.2, 4.7 Hz, 3H), 3.21 (td, *J =* 9.1, 5.7 Hz, 1H), 2.69 (qt, *J =* 9.6, 5.2 Hz, 4H), 2.26 (s, 3H), 2.11 (s, 3H), 1.99 (s, 2H), 1.78 (d, *J =* 4.9 Hz, 3H), 1.51 (s, 3H), 1.18 (t, *J =* 7.1 Hz, 2H), 1.03 (d, *J =* 10.2 Hz, 6H). ^13^C-NMR (100 MHz, ) δ 202.83, 171.86, 171.16, 170.79, 170.13, 169.41, 169.23, 167.04, 167.00, 165.69, 139.84, 137.73, 134.74, 133.96, 133.84, 131.96, 130.41, 130.05, 129.17, 128.81, 128.09, 127.93, 95.06, 93.02, 84.08, 80.75, 78.36, 77.21, 76.69, 75.77, 75.58, 75.19, 75.12, 74.97, 73.46, 72.89, 71.28, 71.08, 70.88, 69.69, 60.83, 60.22, 57.85, 54.36, 46.56, 43.43, 40.60, 40.39, 40.18, 39.98, 39.77, 39.56, 39.35, 37.00, 34.89, 28.91, 28.57, 26.83, 23.02, 21.87, 21.22, 21.14, 14.55, 14.38, 10.25. ESI-MS: *m*/*z* 1138.05 [M + Na]^+^. ^1^H-NMR and ^13^C-NMR spectra of SG-PTX can be seen in Figure S3 and S4.

### 4.3. Synthesis of DG-PTX

Synthesis of C2′-TES paclitaxel (**6**): the mixture of paclitaxel (256.2 mg, 0.3 mmol) and imidazole (40.8 mg, 0.6 mmol) was dissolved in DCM, TESCl (90.4 mg, 100 μL) was added dropwise. The reaction was stirred at r.t. for 4 h, then concentrated under pressure. The residue was purified by silica gel chromatography (Hex:EA = 3:1) and the solvent was removed under pressure to produce a white solid **6** (190.7 mg, yield 66%). ^1^H-NMR (400 MHz, Chloroform-d) δ 8.14 (d, *J =* 7.6 Hz, 2H), 7.74 (d, *J =* 7.7 Hz, 2H), 7.64-7.19 (m, 13H), 7.14 (d, *J =* 8.9 Hz, 1H), 6.29 (d, *J =* 11.1 Hz, 2H), 5.71 (t, *J =* 7.3 Hz, 2H), 5.00–4.96 (m, 1H), 4.70 (d, *J =* 1.9 Hz, 1H), 4.44 (dd, *J =* 11.1, 6.6 Hz, 1H), 4.33 (d, *J =* 8.5 Hz, 1H), 4.23 (d, *J =* 8.4 Hz, 1H), 4.12 (q, *J =* 7.2 Hz, 1H), 3.83 (d, *J =* 7.0 Hz, 1H), 2.60–2.48 (m, 5H), 2.41 (dd, *J =* 15.4, 9.5 Hz, 1H), 2.26–2.13 (m, 4H), 2.04 (s, 2H), 1.94 (d, *J =* 27.7 Hz, 6H), 1.69 (s, 3H), 1.35–1.20 (m, 17H), 1.14 (s, 3H), 0.82 (t, *J =* 8.0 Hz, 9H), 0.45 (ddt, *J =* 19.4, 15.2, 7.5 Hz, 6H).

Synthesis of C7-benzyl glycosylated and C2′-TES paclitaxel (**7**): the mixture of compound **6** (190.7 mg, 0.2 mmol), DMAP (24.4 mg, 0.2 mmol) and DCC (82.5 mg, 0.4 mmol) in THF (2 mL) were added to a solution of compound **3** (256.3mg, 0.4 mmol) in THF (2 mL). After stirring at r.t. in N_2_ atmosphere for 24 h, and concentrating under pressure, the residue was purified by silica gel chromatography (Hex:EA = 7:2), and the solvent was removed under pressure to produce a white solid **7** (164.9 mg, yield 53%). ^1^H-NMR (400 MHz, Chloroform-d) δ 8.12 (s, 2H), 7.74 (s, 2H), 7.52 (s, 4H), 7.29 (s, 27H), 7.14 (s, 3H), 6.36 (s, 1H), 6.23 (s, 1H), 5.67 (dd, *J =* 32.0, 6.8 Hz, 2H), 4.95 (d, *J =* 10.9 Hz, 2H), 4.82 (t, *J =* 10.0 Hz, 3H), 4.68 (d, *J =* 11.9 Hz, 2H), 4.61 (d, *J =* 12.7 Hz, 3H), 4.48 (dd, *J =* 16.5, 11.5 Hz, 3H), 4.33 (d, *J =* 8.4 Hz, 1H), 4.20 (d, *J =* 8.4 Hz, 1H), 3.91 (dq, *J =* 25.2, 8.6, 8.1 Hz, 3H), 3.70 (dt, *J =* 35.9, 10.1 Hz, 6H), 2.78–2.48 (m, 9H), 2.26–1.55 (m, 21H), 1.23 (dq, *J =* 35.9, 17.0, 14.4 Hz, 28H), 0.80 (d, *J =* 8.2 Hz, 9H), 0.43 (dh, *J =* 23.6, 7.8 Hz, 6H).

Synthesis of C7-benzyl glycosylated paclitaxel (**8**): the mixture of compound **7** (164.9 mg, 0.07 mmol) and TBAF (1 mol/L solution in THF, 124 μL, 0.12 mmol) in THF (2 mL) was stirred at r.t. for 6 h and concentrated under pressure. The residue was purified by silica gel chromatography (Hex:EA = 5:2) and the solvent was removed under pressure to produce a white solid **8** (102.4 mg, yield 66%). ^1^H-NMR (400 MHz, Chloroform-d) δ 8.04 (d, *J =* 7.0 Hz, 2H), 7.69 (d, *J =* 7.4 Hz, 2H), 7.47–7.17 (m, 29H), 7.04 (dd, *J =* 20.4, 7.4 Hz, 3H), 6.29 (s, 1H), 6.11 (s, 2H), 5.74 (s, 1H), 5.59 (d, *J =* 6.7 Hz, 1H), 5.53–5.45 (m, 1H), 4.84 (dd, *J =* 22.9, 10.1 Hz, 2H), 4.75 (dd, *J =* 10.6, 7.6 Hz, 3H), 4.64–4.51 (m, 3H), 4.41 (dd, *J =* 14.9, 11.4 Hz, 2H), 4.23 (d, *J =* 8.3 Hz, 1H), 4.10 (d, *J =* 8.3 Hz, 1H), 3.89–3.77 (m, 3H), 3.69 (d, *J =* 9.7 Hz, 2H), 3.62–3.55 (m, 2H), 2.57 (d, *J =* 6.7 Hz, 2H), 2.29 (s, 4H), 2.05 (s, 3H), 1.74 (s, 8H), 1.29–1.03 (m, 28H).

Synthesis of C7 & C2′-benzyl glycosylated paclitaxel (**9**): the mixture of compound **8** (102.4 mg, 0.07 mmol), DMAP (17.1 mg, 0.14 mmol) and DCC (42.9 mg, 0.21 mmol) in THF (3 mL) were added into a solution of compound **3** (89.1 mg, 0.14 mmol) in THF (2 mL). After stirring at r.t. in N_2_ atmosphere for 24 h, and concentrated under pressure, the residue was purified by silica gel chromatography (Hex:EA = 9:2), and the solvent was removed under pressure to produce a white solid **9** (79.3 mg, yield 55%).^1^H-NMR (400 MHz, Chloroform-d) δ 8.14 (d, *J =* 7.5 Hz, 2H), 7.77 (d, *J =* 7.4 Hz, 2H), 7.61–7.10 (m, 53H), 7.00 (d, *J =* 9.1 Hz, 1H), 6.38–6.14 (m, 4H), 5.98 (d, *J =* 9.5 Hz, 1H), 5.72–5.48 (m, 4H), 4.94–4.27 (m, 18H), 4.15 (dd, *J =* 21.3, 7.9 Hz, 1H), 3.99–3.51 (m, 13H), 3.00 (s, 1H), 2.66 (qd, *J =* 12.3, 11.1, 5.4 Hz, 7H), 2.45 (s, 3H), 2.13–1.60 (m, 20H), 1.22–1.08 (m, 7H).

Synthesis of C7 and C2′-double glycosylated paclitaxel (10, DG-PTX): compound **9** (79.3 mg, 0.04 mmol) was dissolved in AcOH (2 mL), then Pd/C (51.1 mg, 0.48 mmol) was added. The reaction was stirred at r.t. in a H_2_ atmosphere for 24 h. After filtration and concentration, the residue was purified by silica gel chromatography (EA:MeOH = 10:1), and the solvent was removed under pressure to produce a white solid **10** (44.3 mg, yield 85%). ^1^H-NMR (400 MHz, DMSO-*d*_6_) δ 9.28 (d, *J =* 8.2 Hz, 1H), 7.99 (d, *J =* 7.6 Hz, 2H), 7.87 (d, *J =* 7.5 Hz, 2H), 7.77–7.66 (m, 3H), 7.59–7.42 (m, 8H), 7.21 (s, 1H), 6.03 (s, 1H), 5.94 (s, 1H), 5.84 (s, 1H), 5.54 (t, *J =* 8.9 Hz, 1H), 5.46–5.35 (m, 3H), 5.16–4.90 (m, 8H), 4.75 (s, 1H), 4.56–4.47 (m, 2H), 4.05 (s, 2H), 3.68–3.43 (m, 11H), 3.19 (d, *J =* 13.5 Hz, 4H), 2.63 (d, *J =* 49.6 Hz, 8H), 2.26 (s, 3H), 2.11 (s, 3H), 1.68 (d, *J =* 25.7 Hz, 8H), 1.24 (s, 2H), 1.01 (d, *J =* 18.5 Hz, 6H). ^13^C-NMR (100 MHz, DMSO-*d*_6_) δ 202.16, 171.94, 171.40, 171.33, 171.22, 171.03, 170.38, 169.58, 169.44, 167.02, 165.68, 140.56, 137.75, 134.73, 134.11, 132.93, 131.99, 130.21, 130.07, 129.22, 128.84, 128.08, 127.90, 127.90, 95.05, 94.93, 93.00, 92.84, 83.29, 80.22, 78.36, 78.26, 77.12, 75.58, 75.13, 74.51, 73.42, 72.88, 72.83, 71.72, 71.09, 71.04, 69.86, 69.70, 69.63, 60.95, 60.83, 60.76, 56.50, 55.66, 54.50, 49.07, 46.53, 43.39, 40.57, 40.36, 40.15, 39.94, 39.73, 39.52, 39.32, 34.72, 33.12, 29.23, 29.18, 28.85, 28.52, 26.70, 23.01, 21.73, 20.97, 19.02, 14.46, 11.11. ESI-MS: *m*/*z* 1379.29 [M + H]^+^. ^1^H-NMR and ^13^C-NMR spectra of DG-PTX can be seen in Figures S5 and S6.

### 4.4. HPLC Determination Assays

The concentration of PTX and PTX prodrugs was determined by HPLC (Waters e2695 HPLC, with a 2998 UV detector, Milford, MA, USA), a C18 column (Lubex, 5μm, 4.6 × 250 mm, Guangzhou, China) was also used. The flow rate was 1 mL/min, with the mobile phase starting at 50% acetonitrile and 50% water and transitioning to 60% acetonitrile and 40% water at 13 min, then returning to 50% acetonitrile and 50% water at 25 min. Chromatographic peaks of the three compounds were identified based on their retention times and chromatographic responses. Peak area measurements were utilized to regress calibration curves to determine each compound.

### 4.5. Solubility Measurements

Excess powder of PTX and PTX prodrugs was added to three kinds of commonly-used media for injection: pure water, 5% glucose solution, and saline, respectively. The test system was shaken at 25 °C for 72 h, then centrifugated at 10,000 rpm for 10 min. Each supernate was collected and 0.22 μm filtered off, then determined by HPLC.

### 4.6. Determination of Log P

Log P and was determined by a miniaturized shake flask method. Briefly, the organic phase consisted of n-octanol saturated with water. Respectively, the aqueous phase consisted of water saturated with n-octanol. Excess powder of PTX prodrug was added and the system was shaken for 24 h at 500 rpm, then centrifuged at 14,000 rpm for 20 min to separate the aqueous and organic layer [28]. The concentration of PTX prodrugs in the organic and aqueous phase was determined by HPLC, each sample was of 0.22 μm filtered off before injection.

### 4.7. Parent Drug Release in Serum

An amount of 5.6 mg of SG-PTX or 6.9 mg of DG-PTX (4.3 mg of PTX equiv.) was dissolved in methanol (0.5 mL), then filled with FBS or RS to 10 mL and the system was incubated at 37 °C with shaking. Then 0.5 mL samples of the system were collected at different times (1, 2, 4, 6, 8, 12, 24 h), then extracted with EA, the organic phase was dried in nitrogen [21,49]. The residue was dissolved in acetonitrile and 0.22 μm filtered off, then determined by HPLC.

### 4.8. Enzymatic Hydrolysis

SG-PTX (5.6 mg) or DG-PTX (6.9 mg) was dissolved in methanol (0.5 mL), then filled up with PBS solution of β-glucuronidase to 10 mL (final concentration of β-glucuronidase was 1140 U/mL). The system was incubated at 37 °C with shaking. Then 0.5 mL of the system was collected at different times (1, 2, 4 h) [50], then extracted with EA, the organic phase was dried in nitrogen. The residue was dissolved in acetonitrile and 0.22 μm filtered off, then determined by HPLC.

### 4.9. Cell Culture

The MDA-MB-231 cell line was supplied by JENNIO Biological Technology (Guangzhou, China). The cells were cultured in L-15 medium (with 10% FBS) in 5% CO_2_ atmosphere in a 37°C incubator.

### 4.10. MTT Assays

MDA-MB-231 cells were seeded in a 96-well transparent plate, at a density of 5000 cells/well. After incubation for 24 h, various concentrations of PTX and PTX prodrugs solution were added, the concentration of PTX and PTX prodrugs ranged from 1 nM to 1000 nM. After 72 h incubation, the plates were washed with HBSS and MTT solution was added. The final concentration of MTT in each well was 0.5 mg/mL. Then 4 h later, the solution on the plate was removed and the content was dissolved with DMSO [33].The absorption was measured at 490 nm and cell viability was calculated, cell viability% = OD of treated cells/OD of untreated cells × 100% [51].

### 4.11. Annexin V-FITC/PI Assays

MDA-MB-231 cells were seeded in a 6-well transparent plate, at a density of 5 × 10^5^ cells/well. After incubation for 24 h, 1 μM of PTX or PTX prodrug solution was added. After 24 h incubation, the cells were harvested by trypsin, washed with HBSS, and 1 mL of binding buffer (containing Annexin V-FITC and PI) was added, final concentration of Annexin V-FITC and PI were both 1 μg/mL [52]. After that, the test system was incubated in the dark for 10 min, then analysis was immediately conducted by flow cytometry. The fluorescence intensity of Annexin V-FITC was measured at an excitation/emission wavelength of 488/530 nm, the fluorescence intensity of PI was measured at an excitation/emission wavelength of 488/617 nm. Each assay was repeated in triplicate.

### 4.12. Solubility of SG-PTX at Various pH Conditions

An aqueous solution of different pH was adjusted by HCl or NaOH, Excess powder of SG-PTX was added to solutions of different pH, respectively. The test system was shaken at 25 °C for 72 h, then centrifugation at 10,000 rpm for 10 min. Each supernate was collected and 0.22 μm filtered off, then determined by HPLC.

### 4.13. Solubility of SG-PTX in Various Formulations

Solutions of different surfactants (Cremophor EL, Tween-80, and PEG-400) were prepared at different concentrations of 2.5%, 5%, 10%, and 15% (*v*/*v*). Excess powder of SG-PTX was added into different formulations, respectively. The test system was shaken at 25 °C for 72 h, then centrifugation at 10,000 rpm for 10 min. Each supernate was collected and 0.22 μm filtered off, then determined by HPLC.

## 5. Conclusions

In conclusion, two glycosylated paclitaxel prodrugs were synthesized, both of the two prodrugs significantly increased the solubility of PTX and manifested suitable drug-like lipophilicity properties. Both of the two prodrugs can release free PTX in serum and be hydrolyzed by the action of β-glucuronidase. The prodrugs also maintain effective cytotoxicity and competitive apoptosis inducing effect against breast cancer cells. Moreover, on comparing with DG-PTX, SG-PTX presents a much better release and hydrolyzing degree. It is envisaged that this glycosylated strategy is viable for PTX prodrug design while SG-PTX is a feasible and potential type of PTX prodrug, based on its solubility, lipophilicity, anti-cancer effect, drug releasing behavior, and enzymatic hydrolyzing degree.

The highly soluble formulation of SG-PTX by Cremophor EL developed in this study can be a feasible and effective alternative to paclitaxel formulation. Because SG-PTX presented a promising solubility in a low percentage of an ethanol-free Cremophor EL solution, this helps to reduce the dosage of toxic surfactant making it safer than the current commercial formulation.

## Figures and Tables

**Table 1:** Solubility (μg/mL) of SG-PTX, DG-PTX, and PTX. All values are expressed as mean ± SD. (n = 4). “SG-PTX” stands for single glucose-conjugated PTX prodrug, “DG-PTX” for double glucose-conjugated PTX prodrug and “PTX” for paclitaxel.

Compound	Pure Water	5% Glucose	Saline
SG-PTX	19.93 ± 4.07	17.85 ± 0.28	17.83 ± 0.09
DG-PTX	38.97 ± 5.66	34.78 ± 4.22	36.72 ± 2.05
PTX	<1	<1	<1

**Table 2:** Log P determination of SG-PTX, DG-PTX, and PTX. All values are expressed as mean ± SD (n = 4).

Compound	Log P
SG-PTX	2.77 ± 0.16
DG-PTX	2.12 ± 0.08
PTX	3.97 [27]

## Supplementary Materials

The following are available online. Figure S1: Linearity calibration of SG-PTX, Figure S2: Linearity calibration of DG-PTX, Figure S3: ^1^H-NMR spectra of SG-PTX, Figure S4: ^13^C-NMR spectra of SG-PTX, Figure S5: ^1^H-NMR spectra of DG-PTX, Figure S6: ^13^C-NMR spectra of DG-PTX.
