# Supplementary material for: Synthesis, Biological Evaluation and Low-Toxic Formulation Development of Glycosylated Paclitaxel Prodrugs
Source: Molecules. 2018 Dec 5;23(12):3211. doi: 10.3390/molecules23123211 (PMC6321537; doi:10.3390/molecules23123211)
Supplement: Supplementary file 1 [file molecules-23-03211-s001.pdf]

Synthesis, Biological Evaluation, and Low-Toxic Formulation Development of Glycosylated Paclitaxel Prodrugs

Yukang Mao, Yili Zhang, Zheng Luo, Ruoting Zhan, Hui Xu, Weiwen Chen * and Huicai Huang *

Research Center of Chinese Herbal Resource Science and Engineering, Guangzhou University of Chinese Medicine; Key Laboratory of Chinese Medicinal Resource from *Lingnan* (Guangzhou University of Chinese Medicine), Ministry of Education; Joint Laboratory of National Engineering Research Center for the Pharmaceutics of Traditional Chinese Medicines, Guangzhou, China

Contents

Title Page .... S**Error! Bookmark not defined.**

Linearity calibration of SG-PTX and DG-PTX S2

Spectra S3

Linearity calibration of SG-PTX and DG-PTX

Concentrations of SG-PTX and DG-PTX in samples were calculated by calibration curve. For calibration curve, nine concentrations (9.66, 19.32, 38.41, 76.81, 153.63, 307.25, 614.5, 1229 and 2458 μg/mL of SG-PTX, 9.53, 19.06, 38.12. 76.25, 152.5, 305.5, 611.1, 1220 and 2440 μg/mL of DG-PTX) of calibration standards were processed, typical linear regression equations for the calibration curves over concentration ranges for SG-PTX and DG-PTX were shown in Figure S1 and Figure S2.

Molecules 2018, 23, 3211; doi:10.3390/molecules23123211

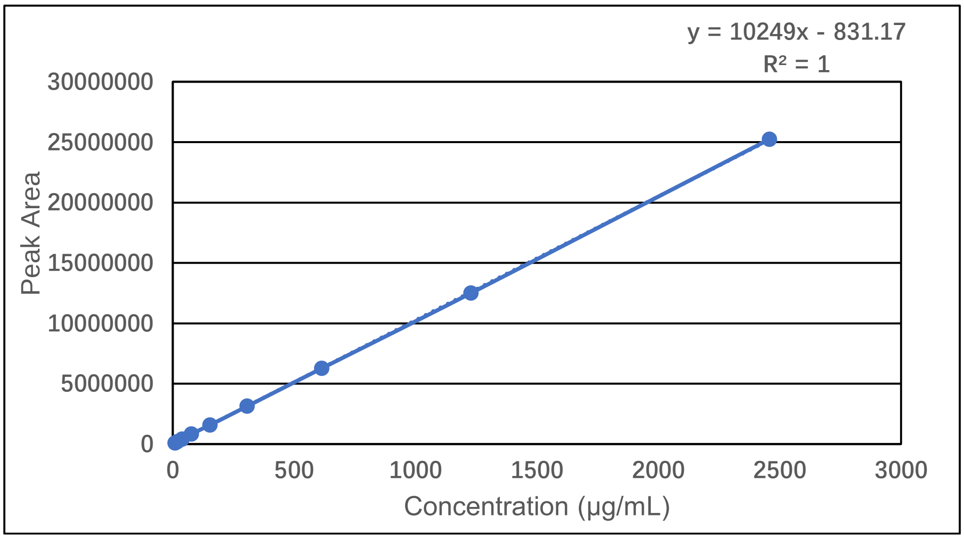

Figure S1. Linearity calibration of **SG-PTX.**

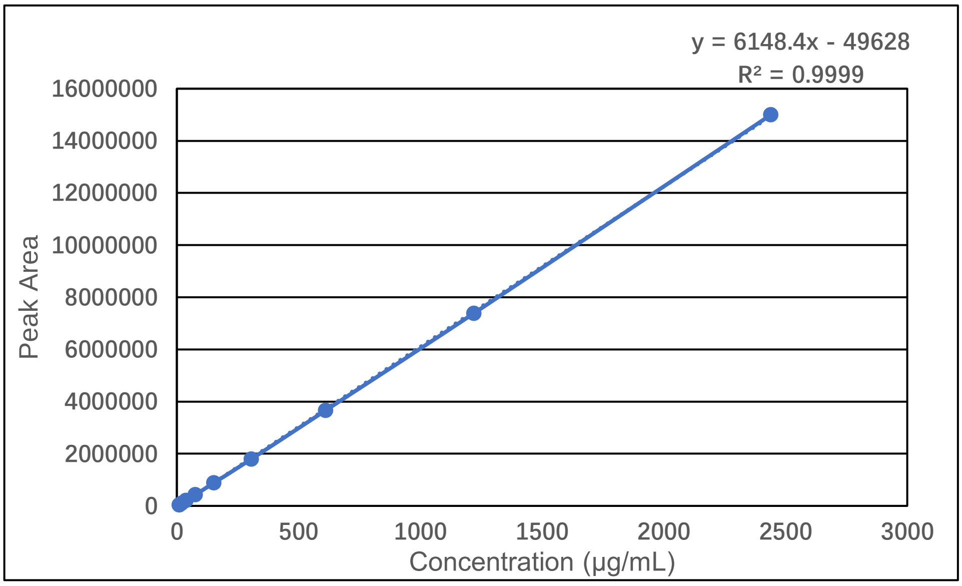

Figure S2. Linearity calibration of **DG-PTX.**

Molecules 2018, 23, 3211; doi:10.3390/molecules23123211

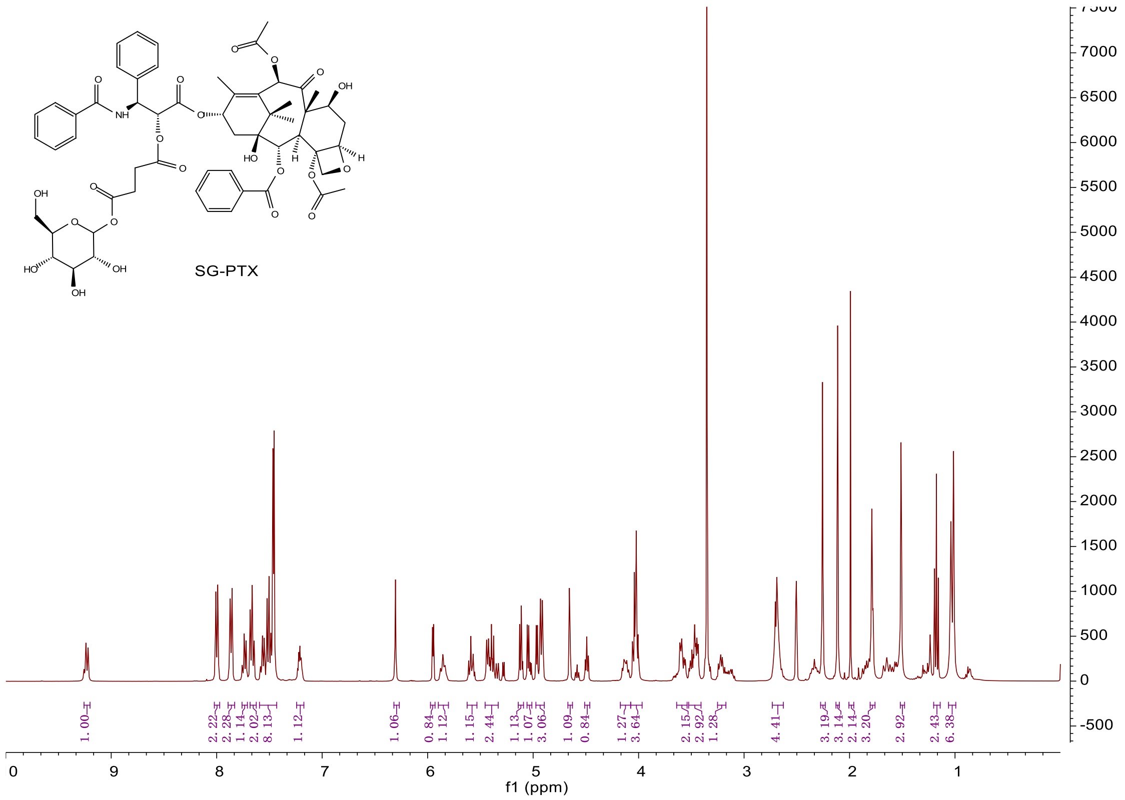

Figure S3. ^{1}H NMR spectra of **SG-PTX.**

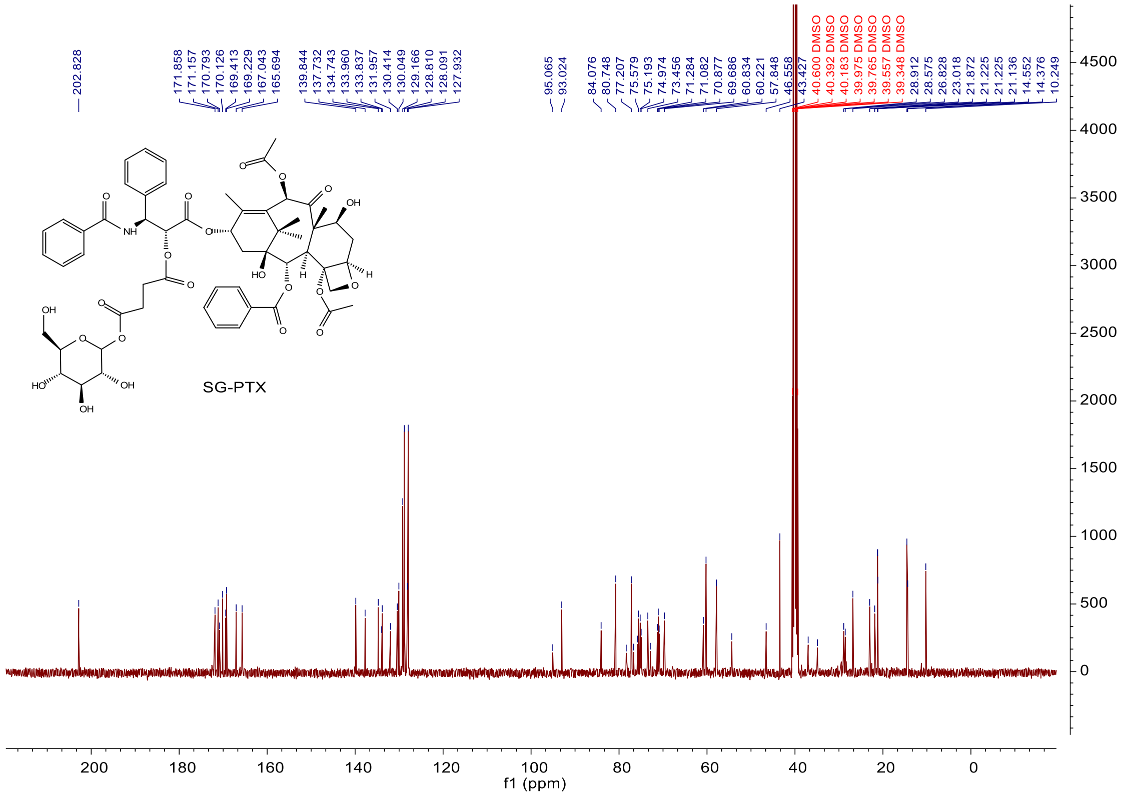

Figure S4. ^{13}C NMR spectra of **SG-PTX.**

Molecules 2018, 23, 3211; doi:10.3390/molecules23123211

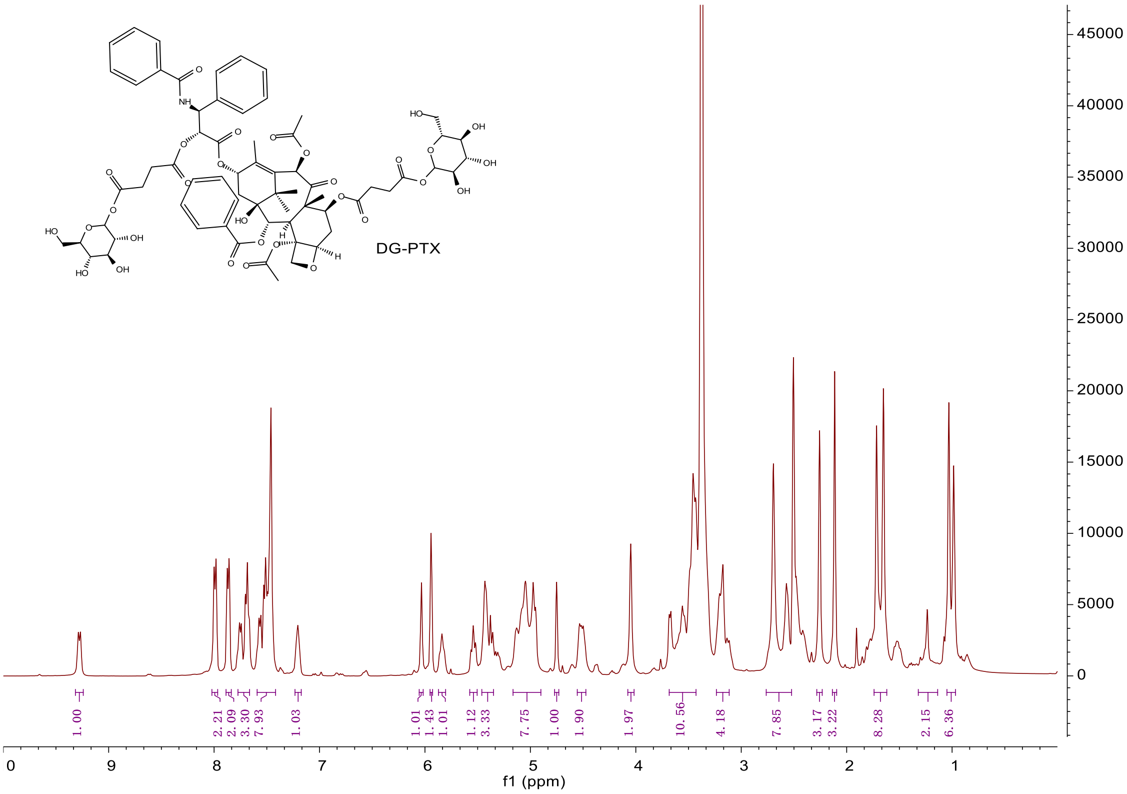

Figure S5. ^{1}H NMR spectra of **DG-PTX.**

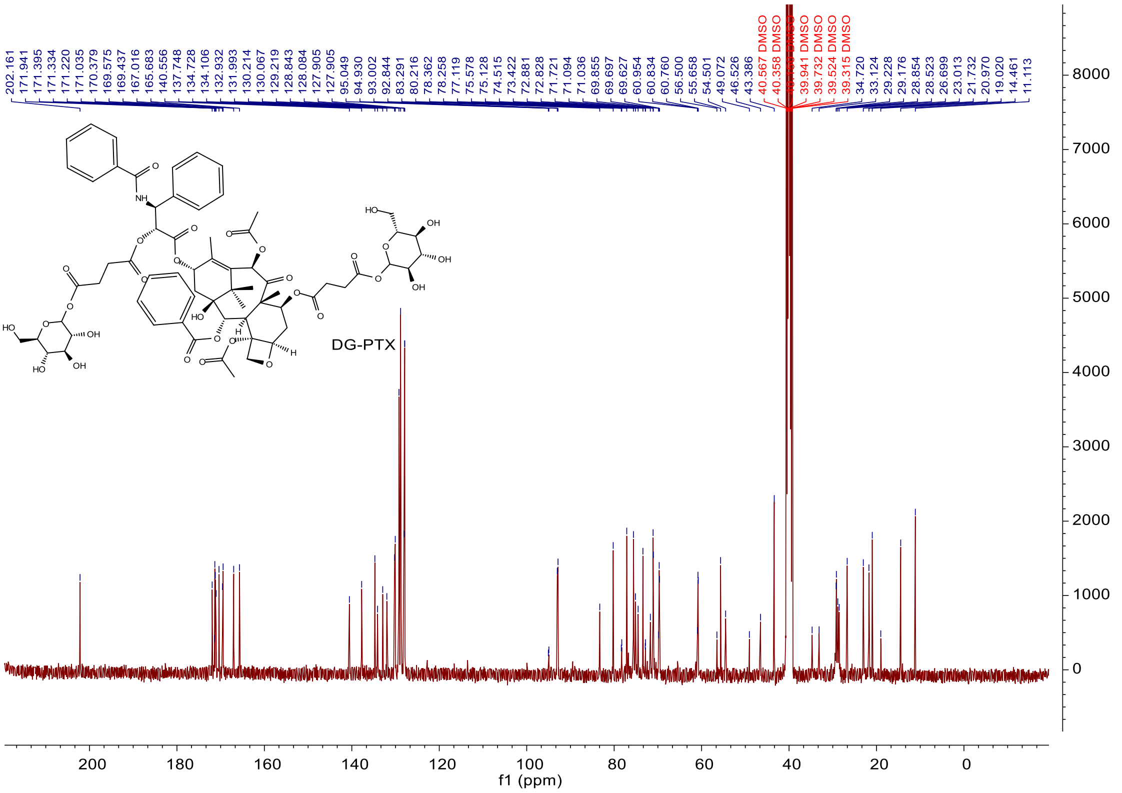

Figure S6. ^{13}C NMR spectra of **DG-PTX.**

Molecules 2018, 23, 3211; doi:10.3390/molecules23123211
